# Editorial: Basic and clinical research on adult snoring and obstructive sleep apnea syndrome

**DOI:** 10.3389/fneur.2023.1246928

**Published:** 2023-08-03

**Authors:** Yewen Shi, Xiangdong Tang, Tongxin Xie, Yitong Zhang, Xiaoyong Ren

**Affiliations:** ^1^Department of Otorhinolaryngology-Head and Neck Surgery, The Second Affiliated Hospital of Xi'an Jiaotong University, Xi'an, Shaanxi, China; ^2^Department of Respiratory and Critical Care Medicine, Sleep Medicine Center, Mental Health Center, Translational Neuroscience Center, Chengdu, China; ^3^State Key Laboratory of Biotherapy, West China Hospital, Sichuan University, Chengdu, China; ^4^Department of Head and Neck Surgery, The University of Texas MD Anderson Cancer Center, Houston, TX, United States

**Keywords:** obstructive sleep apnea syndrome, genetic heterogeneity, animal model, continuous positive airway pressure, drug-induced sleep endoscopy

Obstructive sleep apnea (OSA) is characterized by repeated episodes of upper airway closure during sleep, resulting in recurrent oxyhemoglobin desaturation and sleep fragmentation (1). By 2020, the prevalence of OSA in the United States reached 8.7–43.2% (2). The main clinical symptoms of OSA include snoring, nocturnal awakening, nocturia, unrefreshing sleep, and daytime sleepiness, resulting in reduced quality of life. If without treatment, OSA can also increase the risk for systemic complications such as cognitive impairment, Alzheimer's disease, atrial fibrillation, stroke, type 2 diabetes, and so on (3). Therefore, increasing academic attention to OSA disease and achieving early prevention, diagnosis, and treatment can effectively avoid related complications and reduce the burden on the global healthcare system. This Research Topic mainly focuses on bioinformatics studies, animal studies, and clinical studies of treatment on OSA and its comorbidities, aiming to guide the academic discussion, strengthen international cooperation, and promote OSA research.

OSA is an individually variable disease with multiple symptoms and endogenous types. More and more studies have recognized the importance of individual differentiation and genetic heterogeneity in the management of OSA and its complications. The advent of bioinformatic methods has led to great advances in the pathophysiology and mechanisms studies of OSA. Chronic intermittent hypoxia (CIH), described as repeated hypoxia and reoxygenation, is a unique pathological mechanism of OSA that aggravates body damage. CIH can cause an increase in reactive oxygen species (ROS), which may lead to cellular ferroptosis. Based on the relationship between oxidative stress and ferroptosis, Liu et al. identified HIF1A and ATM as key genes responsible for ferroptosis during CIH using bioinformatics and animal experiments. They found that HIF1A causes increased oxidative stress and general inflammation, in addition to regulating a variety of glycolysis, proliferation, invasion, and survival genes during the hypoxic response. ATM is a type of protein kinase that is essential for both cellular inflammatory toxicity and oxidative stress-induced cell death. Central genes may be used as biomarkers and treatment targets for OSA.

Cardiometabolic diseases are the most complex complication of OSA. Ding et al. assessed the gene-predicted associations of OSA with type 2 diabetes (T2D), non-alcoholic fatty liver disease (NAFLD), and coronary heart disease (CHD) based on the mendelian randomization method. Outcomes excluded genetically predicted linkages of OSA with T2D, NAFLD, and CHD risk, and indicated that genes associated with obesity might obfuscate prior observations. The possibility that obesity may be a critical element in linking OSA to cardiometabolic diseases should be taken into account in subsequent clinical and scientific research studies.

Further studies at the genetic, molecular, and cellular levels need to be validated in animal models designed to mimic the pathophysiological characteristics of OSA patients. Zong et al. reviewed the typical animal models of OSA and separately analyzed their characteristics. In specific studies, existing animal models can be selected and modified depending on the study design. Evaluating the reliability of animal models of OSA includes PSG, upper airway imaging, and electronic nasopharyngoscopy of small animals.

Continuous positive airway pressure (CPAP) and surgery, primarily uvulopalatopharyngoplasty (UPPP), are the first-line therapy for OSA and its complications. The selection and efficacy of individualized treatment for patients with OSA have always been the focus. Cao et al. assessed the value of drug-induced sleep endoscopy (DISE), a novel technology, in predicting the prognosis of surgery in patients with OSA. They found that the lateral collapse of the velopharyngeal airway, without consideration of hypopharyngeal collapse, and minor hypopharyngeal collapse in patients with anteroposterior collapse or concentric collapse of the velopharyngeal airway may be predictive of a superior operative result. Patients with lateral velopharyngeal airway collapse and grade I glossopharyngeal airway collapse degree are suitable for velopharyngeal surgery.

OSA is related to an increased risk of all-cause and cardiovascular mortality, as well as the incidence of postoperative cardiovascular events (4). OSA is prevalent in patients undergoing cardiac valve replacement (CVR). OSA contributes to the disease burden of CVR, including an increased mechanical ventilation duration and a longer intensive care unit (ICU) stay. The benefits of CPAP for patients with OSA and CVR are unclear. Su et al. evaluated the impact of perioperative 1-week CPAP therapy on postoperative cardiopulmonary prognosis in patients with OSA combined with valvular heart disease. In patients undergoing CVR, the use of preoperative auto-CPAP for OSA resulted in a statistically significant reduction in mechanical ventilation time and postoperative length of stay in the ICU and hospital. However, it was not related to postoperative arrhythmias, pacemaker use, reintubation, or pneumonia. In follow-up studies, more questions need to be explored, for example, what is the appropriate length of CPAP treatment for OSA patients undergoing CVR surgery? All questions deserve in-depth research and discussion in the future.

Multidisciplinary, precise, and individualized treatment is the trend in OSA diagnosis and treatment. At present, the research on OSA and multi-system diseases has shown a 100 flowers and a 100 schools of thought. Innovative advances have been made in related animal experiments, bioinformatics, and clinical treatment strategies. At the same time, the new advances in mechanism and clinical research are of great significance to the updating of concepts and the improvement of clinical diagnosis and treatment techniques. In clinical work, we should pay attention to the screening of OSA-related comorbidities and take measures to reduce or mitigate the risk of the disease to improve patient satisfaction and the quality of OSA diagnosis and treatment.

## Author contributions

YS and YZ prepared the original draft. XR, XT, and TX critically reviewed and edited the manuscript. All authors have reviewed and approved the final manuscript.
